# SRSF1 Deficiency Impairs the Late Thymocyte Maturation and the CD8 Single-Positive Lineage Fate Decision

**DOI:** 10.3389/fimmu.2022.838719

**Published:** 2022-01-26

**Authors:** Ce Ji, Li Bao, Shunzong Yuan, Zhihong Qi, Fang Wang, Menghao You, Guotao Yu, Jingjing Liu, Xiao Cui, Zhao Wang, Juanjuan Liu, Wenhui Guo, Mingxia Feng, Feng Chen, Youmin Kang, Shuyang Yu

**Affiliations:** ^1^ State Key Laboratory of Agrobiotechnology, College of Biological Sciences, China Agricultural University, Beijing, China; ^2^ Department of Hematology, Beijing Jishuitan Hospital, Beijing, China; ^3^ Department of Hematology, The Fifth Medical Center of People’s Liberation Army (PLA) General Hospital, Beijing, China; ^4^ Central Laboratory, School of Stomatology, Peking University, Beijing, China

**Keywords:** SRSF1, thymocyte, lineage choice, CD8^+^ T cell, Runx3, development

## Abstract

The underlying mechanisms of thymocyte development and lineage determination remain incompletely understood, and the emerging evidences demonstrated that RNA binding proteins (RBPs) are deeply involved in governing T cell fate in thymus. Serine/arginine-rich splicing factor 1 (SRSF1), as a classical splicing factor, is a pivotal RBP for gene expression in various biological processes. Our recent study demonstrated that SRSF1 plays essential roles in the development of late thymocytes by modulating the T cell regulatory gene networks post-transcriptionally, which are critical in response to type I interferon signaling for supporting thymocyte maturation. Here, we report SRSF1 also contributes to the determination of the CD8^+^ T cell fate. By specific ablation of SRSF1 in CD4^+^CD8^+^ double positive (DP) thymocytes, we found that SRSF1 deficiency impaired the maturation of late thymocytes and diminished the output of both CD4^+^ and CD8^+^ single positive T cells. Interestingly, the ratio of mature CD4^+^ to CD8^+^ cells was notably altered and more severe defects were exhibited in CD8^+^ lineage than those in CD4^+^ lineage, reflecting the specific function of SRSF1 in CD8^+^ T cell fate decision. Mechanistically, SRSF1-deficient cells downregulate their expression of *Runx3*, which is a crucial transcriptional regulator in sustaining CD8^+^ single positive (SP) thymocyte development and lineage choice. Moreover, forced expression of Runx3 partially rectified the defects in SRSF1-deficient CD8^+^ thymocyte maturation. Thus, our data uncovered the previous unknown role of SRSF1 in establishment of CD8^+^ cell identity.

## Introduction

T cell development occurs in the thymus and consists of several ordered processes, such as T cell lineage commitment, T cell receptor (TCR) rearrangements, expression of diverse TCR repertoire, positive and negative selection, and the terminal maturation for acquisition of their functions as helper, cytotoxic or regulatory T cells (1–4). A lymphoid precursor developing into a mature αβT cell undergoes three major sequential phases defined by the CD4 and CD8 expression, including CD4^−^CD8^−^ double negative (DN), CD4^+^CD8^+^ double positive (DP), and either CD4^+^CD8^−^ or CD4^−^CD8^+^ single positive (SP) stages (2, 5). The dynamic expression of cell surface markers which are related to functional alterations is essential to delineate the stages of thymocyte development (6). For instance, the thymocytes are stratified into distinct developmental stages defined by the expression of TCRβ (or CD3e) and the activation marker CD69, representing preselection (TCRβ^lo^CD69^lo^), initial stage of selection (TCRβ^int^CD69^lo^), undergoing selection (TCRβ^int^CD69^hi^), post selected immature (TCRβ^hi^CD69^hi^), and post selected mature (TCRβ^hi^CD69^lo^) thymocytes, respectively (7–9). In addition, SP thymocytes are also a heterogeneous population which gradually proceed to downregulate heat-stable antigen (HSA, CD24) and upregulate Qa2 before entry into the periphery T cell pool (6, 10). Hence, the post selected TCRβ^hi^ thymocytes can be further compartmentalized by the dynamic expression level of CD69, CD24, CD4 and CD8 on their cell surface, reflecting the heterogeneity and defining the developmental stages of late thymocytes (11, 12).

DP thymocytes first express the mature αβTCR complex which allows the engagement by intrathymic peptide major histocompatibility complex (MHC) ligands and interact with stromal cells that are localized in the cortex for positive and negative selection (13). After positive selection, DP cells expressing MHC class I- or MHC class II-TCRs selectively differentiate into either conventional CD4^+^ helper or CD8^+^ cytotoxic T cells, which is a critical developmental event known as the CD4/CD8 lineage choice. Based on the theory of the kinetic signaling model, most of positively selected DP thymocytes must pass through an intermediate CD4^+^CD8^lo^ stage and both duration and intensity of TCR signaling exert essential impact on cell fate decision (14). To comprehend the underlying intracellular mechanisms involved in the CD4/CD8 lineage commitment, a few transcription factors have been identified, such as Thpok, Runx3, Mazr, Myb, Bcl11b, Gata3, Tox, Tcf1/Lef1, and Tle factors (11, 12, 15–21). Among them, Thpok and Runx3 are critical for specification of CD4^+^ helper or CD8^+^ cytotoxic cells, respectively, and play central roles in controlling CD4/CD8 lineage choice (22). To date, a complete understanding of the process awaits elucidation of the precise mechanisms involved in the extensive regulatory network.

The RNA-binding protein serine/arginine splicing factor 1 (SRSF1, also named ASF/SF2) belongs to the highly conserved SR protein family which functions as a key regulator in most cell types *via* mediating mRNA metabolism, such as constitutive and alternative splicing, RNA polymerase II transcription, nuclear export of mature mRNA and translation, and genomic stability (23–27). Our recent studies have demonstrated that SRSF1 not only plays a critical role in the late stage development of conventional T cells by controlling the expression of *Il27ra* and *Irf7* transcripts (28), but also serves as an important post-transcriptional regulator in promoting the development and functional differentiation of iNKT cell *via* balancing the abundances of two transcriptional isoforms of Myb (29). These findings suggest that SRSF1 is profoundly involved in the development and function of both conventional and unconventional T cells.

In this study, we employed *Srsf1* ^fl/fl^ *Cd4*-Cre mice to investigate the potential role of SRSF1 in determination of CD4/CD8 lineage fate by specific ablation of SRSF1 in DP thymocytes. The ratio of mature CD4^+^ to CD8^+^ cells was notably altered and more severe defects were exhibited in CD8^+^ lineage, albeit the maturation of both CD4^+^ and CD8^+^ SP T cell was impaired in SRSF1-deficient mice, suggesting the specific function of SRSF1 in CD8^+^ T cell fate decision. Moreover, SRSF1-deficient cells exhibit the reduced abundance of *Runx3* and forced expression of Runx3 partially rectifies the defects in CD8^+^ lineage proportion.

## Results

### Conditional Ablation of SRSF1 at DP Stage Impairs the Maturation of Late Thymocytes

Our recent study has shown that SRSF1 regulates the terminal maturation of thymocytes by post-transcriptionally regulating the abundances of *Il27ra* and *Irf7* functional transcripts *via* alternative splicing (28). By reviewing the phenotype of thymocytes from *Srsf1* ^fl/fl^ *Lck* ^Cre/+^ mice, we found that the numbers of CD8 single-positive (SP) cells are more severe reduction than those of CD4^+^ SP cells, resulting in the substantially altered ratio of CD4^+^ to CD8^+^ cells (**Figures S1A–C**). In addition, we performed gene set enrichment analysis (GSEA) by using our published RNA-seq data (GSE141349). The results indicated that CD8^+^ cell-specific genes were enriched in wild-type DP cells relative to SRSF1-deficient DP cells, suggesting that the differentiation capacity of DP cell toward CD8^+^ SP was more significantly reduced in absence of SRSF1, although both CD4^+^ and CD8^+^ SP thymocyte-related genes exhibited the enrichment in wild-type DP cells (**Figure S1D**). To address the potential role of SRSF1 involved in the lineage choice of CD4-versus-CD8 thymocytes, we established the genetic mouse model with conditional inactivation of SRSF1 in DP stage by crossing *Srsf1* ^fl/fl^ mice with *Cd4*-Cre mice (30), which is widely applied for the lineage determination analysis of late thymocytes (**Figure S2A**). The deletion efficiency of SRSF1 was further confirmed in district subsets along with the sequential developmental phases, indicating the effective deletion of *Srsf1* was achieved in DP and CD4/CD8 SP thymocytes from *Srsf1* ^fl/fl^ *Cd4*-Cre mice compared with those in their littermate control mice (henceforth called Control) (**Figure S2B**).

**Figure 1:**
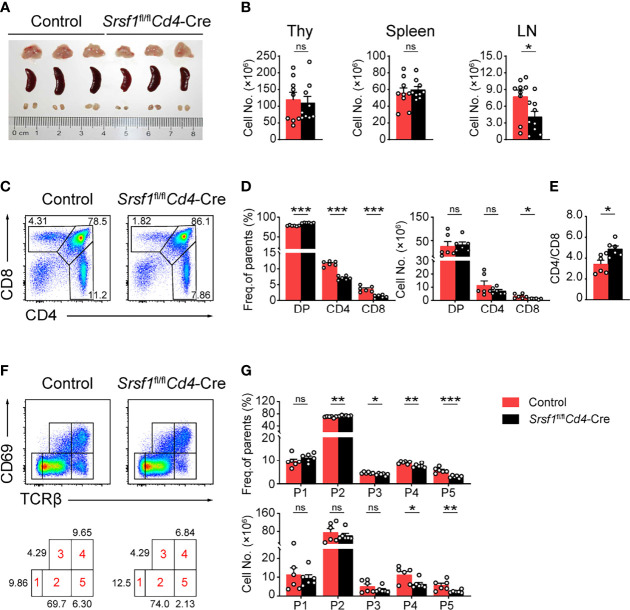
*Srsf1* ^fl/fl^ *Cd4*-Cre mice exhibits defects in the maturation of late thymocytes. **(A)** Images of Thymus (Thy), spleen, and lymph nodes (LNs) from Control and *Srsf1* ^fl/fl^ *Cd4*-Cre mice were shown (n = 3 per group). **(B)** Total cell numbers of Thy, spleen, and LNs from Control and *Srsf1* ^fl/fl^ *Cd4*-Cre mice were shown (n = 9). **(C–E)** Flow cytometry analysis of CD4^+^, CD8^+^, and CD4^+^CD8^+^ double positive (DP) thymocytes. Representative pseudocolor plots show the indicated populations in Control and *Srsf1* ^fl/fl^ *Cd4*-Cre mice in **(C)**, and the frequency and numbers of indicated populations were shown in **(D)**, accordingly. The ratio of frequency between CD4^+^, and CD8^+^ thymocytes was calculated and shown in **(E)** (n = 6). **(F, G)** Flow cytometry analysis of the sequentially developmental stages. **(F)** Representative pseudocolor plots show five subsets, including population 1 (P1: TCRβ^lo^CD69^lo^), population 2 (P2: TCRβ^int^CD69^lo^), population 3 (P3: TCRβ^int^CD69^hi^), population 4 (P4: TCRβ^hi^CD69^hi^), and population 5 (P5: TCRβ^hi^CD69^lo^) in Control and *Srsf1* ^fl/fl^ *Cd4*-Cre mice. The frequency and numbers of indicated subsets were shown in **(G)** (n = 6). Data were collected from at least three independent experiments. The error bars are means ± standard deviation (SD). Statistical significance was determined by one-tailed Student’s *t*-test. ns, not statistically significant; *P < 0.05, **P < 0.01, and ***P < 0.001.

We next analyzed the phenotype of these conditional knock out mice. Compared with their controls, *Srsf1* ^fl/fl^ *Cd4*-Cre mice exhibited comparable size and cellularity of thymus and spleen, but diminished cell number in lymph nodes (**Figures 1A, B**). The frequency of both CD4^+^ and CD8^+^ thymocytes from SRSF1-deficient mice was significantly decreased (**Figures 1C, D**), whereas the percentage of DP thymocytes was correspondingly increased, reflecting a blockade of DP thymocyte development. The cell numbers of CD8^+^ thymocytes in SRSF1-deficient mice were significantly reduced, but no statistical difference in absolute numbers of DP and CD4^+^ thymocytes was observed. The ratio of CD4^+^ cells to CD8^+^ cells was notably altered (**Figure 1E**), implying more severe impacts on CD8^+^ lineage development caused by conditional *Srsf1* deletion in DP thymocytes. To determine the specific developmental stage of thymocytes that was impaired in *Srsf1* ^fl/fl^ *Cd4*-Cre mice, we carved up thymocytes at five distinct developmental phases defined by the expression of TCRβ and the activation marker CD69 as previous described (8, 9, 31) (**Figure 1F**). There was no significant difference observed from populations 1 to 3 between *Srsf1* ^fl/fl^ *Cd4*-Cre mice and their controls, implying the DP thymocytes at pre-selection and the initial stage of positive selection were not affected in absence of SRSF1 (**Figures 1F, G**). In contrast, *Srsf1* ^fl/fl^ *Cd4*-Cre mice had significantly fewer cells in populations 4 to 5 which include post-selected DP, immature SP, and mature SP thymocytes, respectively. These results indicate that ablation of SRSF1 at DP thymocytes mainly impairs the T cell development beyond the post-selection phase.

### SRSF1 Deficiency Alters the Ratio of CD4^+^ to CD8^+^ Cells in TCRβ^hi^ Thymocytes

**Figure 2:**
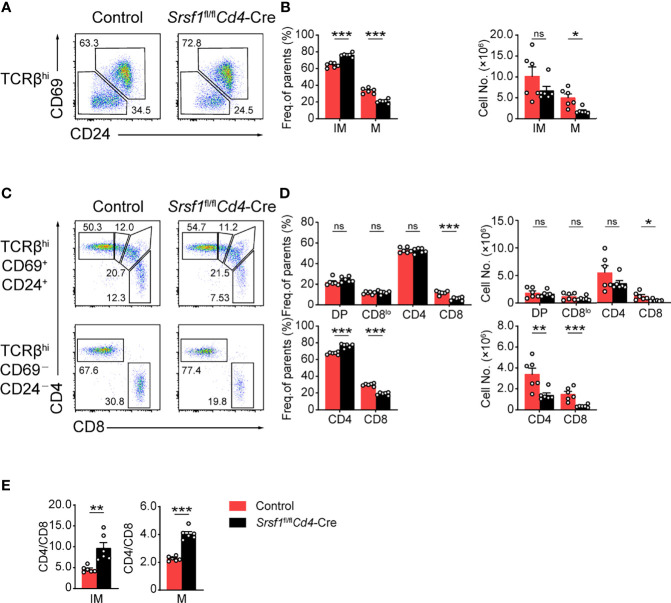
Ablation of SRSF1 severely hinders the maturation of CD8 single-positive thymocytes. **(A–D)** Characterization of the post-selection thymocytes. **(A)** TCRβ^hi^ thymocytes (populations 4 and 5 in **Figure 1F**) were further fractionated into CD69^+^CD24^+^ immature (IM) and CD69^−^CD24^−^ mature (M) subsets. The immature subsets were subdivided into CD4^+^, CD4^+^CD8^lo^ (CD8^lo^), DP, and CD8^+^ sub-populations (clockwise from top left in the top row), and the mature subsets were further subdivided into CD4^+^ and CD8^+^ populations (bottom row) **(C)**. The frequency and numbers of indicated subsets were shown in **(B, D)**, respectively. **(E)** The ratio of CD4^+^ to CD8^+^ thymocytes was calculated and shown (n = 6). Data were collected from at least three independent experiments. The error bars are means ± SD. Statistical significance was determined by one-tailed Student’s *t*-test. ns, not statistically significant; *P < 0.05, **P < 0.01, and ***P < 0.001.

We next focused on the post-selection TCRβ^hi^ thymocytes with an additional maturation marker CD24 staining combined with the activation marker CD69 of thymocytes as previously described (28). The frequency and cell numbers of TCRβ^hi^CD69^-^CD24^-^ mature subset were decreased from *Srsf1* ^fl/fl^ *Cd4*-Cre mice compared with those from Controls (**Figures 2A, B**). The frequency of TCRβ^hi^CD69^+^CD24^+^ immature T cell exhibited a relative increase, but the numbers were slightly diminished (**Figures 2A, B**). By further analysis of the expression of CD4 and CD8 in TCRβ^hi^CD69^+^CD24^+^ immature subsets, we found that the frequency and numbers of DP, CD4^+^CD8^lo^ intermediate cells, and CD4^+^ SP subsets were not significantly alerted, but the frequency and numbers of CD8^+^ SP were remarkably decreased in *Srsf1* ^fl/fl^ *Cd4*-Cre mice (**Figures 2C, D**). In SRSF1-deficient TCRβ^hi^CD69^-^CD24^-^ mature population, the numbers of CD4^+^ and CD8^+^ SP were dramatically diminished, though the frequency of CD4^+^ SP cells was increased whereas the frequency of CD8^+^ SP cells was reduced (**Figures 2C, D**). Moreover, the ratio of CD4^+^ to CD8^+^ SP cells was notably increased in both TCRβ^hi^CD69^+^CD24^+^ immature and TCRβ^hi^CD69^-^CD24^-^ mature thymocytes from *Srsf1* ^fl/fl^ *Cd4*-Cre mice (**Figure 2E**). Collectively, these data indicated that SRSF1 deficiency impaired the terminal maturation of both CD4^+^ and CD8^+^ SP cells, and led to the aberrant ratio of CD4^+^ to CD8^+^ SP cells.

### SRSF1 Deficiency Disturbs the Proportion of CD4^+^ to CD8^+^ Cells in Periphery T Cell Pool

**Figure 3:**
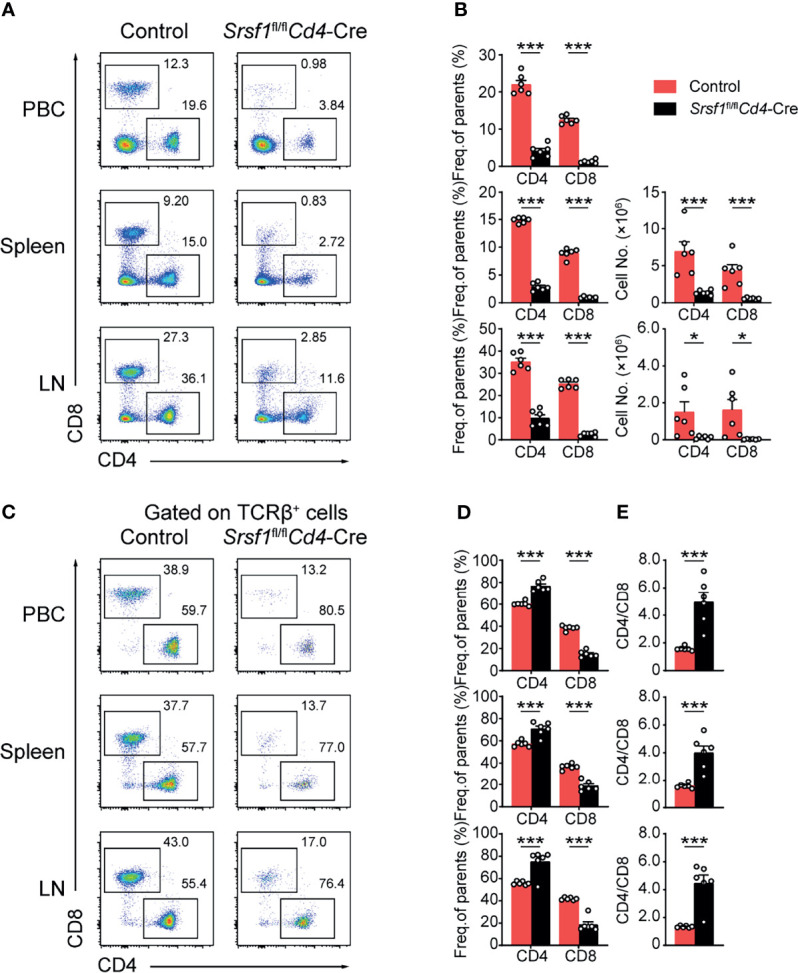
The proportion of peripheral CD4^+^ to CD8^+^ T cells was disturbed in *Srsf1* ^fl/fl^ *Cd4*-Cre mice. **(A, B)** Flow cytometry analysis of T cells in peripheral tissues. **(A)** Representative pseudocolor plots show CD4^+^ and CD8^+^ T cells in PBC, spleen, and LNs from Control and *Srsf1* ^fl/fl^ *Cd4*-Cre mice. The frequency and numbers of indicated subsets in spleen, and LNs were shown in **(B)**, accordingly (*n* = 6). **(C–E)** Analysis of the ratio of frequency between peripheral CD4^+^ T cells and CD8^+^ T cells. **(C)** Representative pseudocolor plots show CD4^+^ and CD8^+^ T cells from TCRβ^+^ populations in PBCs, spleen, and LNs. The frequency and numbers of indicated subsets were shown in **(D)** (*n* = 6), and the ratio of frequency between CD4^+^ T cells to CD8^+^ T cells was calculated and shown in **(E)**, respectively. Data were collected from at least three independent experiments. The error bars are means ± SD. Statistical significance was determined by one-tailed Student’s *t*-test. *P < 0.05 and ***P < 0.001.

We next checked whether the peripheral T cell pool was affected in *Srsf1* ^fl/fl^ *Cd4*-Cre mice. The mature CD4^+^ and CD8^+^ T cell populations in spleens, LNs and PBCs were remarkably diminished in *Srsf1* ^fl/fl^ *Cd4*-Cre mice (**Figures 3A, B**). By further analysis of the proportion of CD4^+^ to CD8^+^ cells in peripheral tissues, we found the frequency of CD4^+^ T cells was increased in SRSF1-deficient TCRβ^+^ cells, and the ratio of CD4/CD8 in peripheral tissues was increased, accordingly (**Figures 3C–E**). These results suggested the critical requirement of SRSF1 in maintaining the numbers of mature T cells, especially CD8^+^ cells in periphery T cell pool.

### SRSF1 Regulates the Maturation of Late Thymocytes in a Cell-Intrinsic Manner

**Figure 4:**
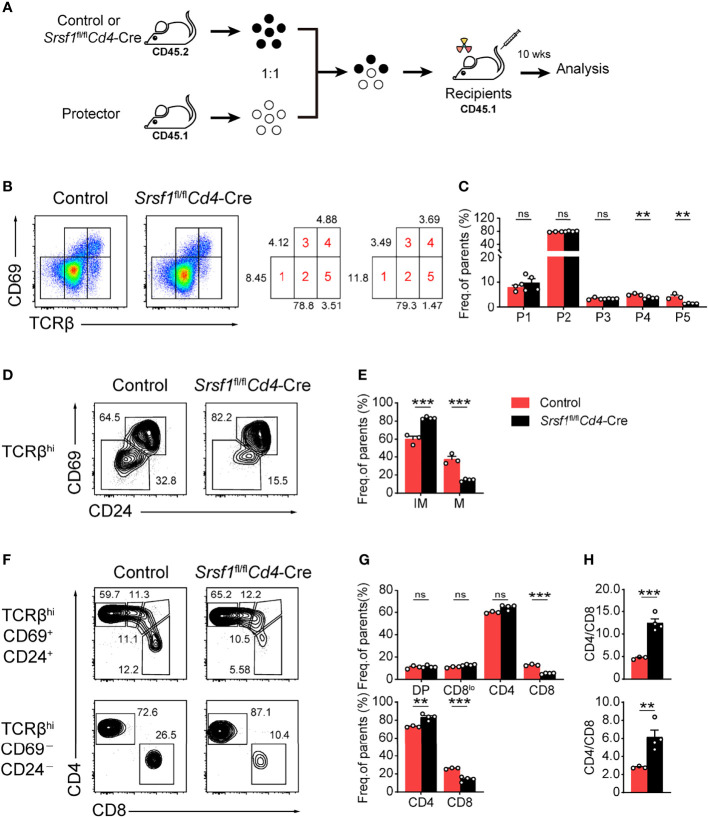
SRSF1 intrinsically regulates the maturation of late thymocytes. **(A)** The scheme of bone marrow chimeric mice generation. A 1:1 mixture of bone marrow cells from Control or *Srsf1* ^fl/fl^ *Cd4*-Cre mice (CD45.2^+^) together with protector bone marrow cells from B6.SJL wild-type (CD45.1^+^) was transplanted into lethally irradiated B6.SJL recipients (CD45.1^+^). The thymocyte development of recipients was analyzed 10 weeks post transplantation. **(B, C)** Flow cytometry analysis of the sequentially developmental stages of donor-derived thymocytes. **(B)** Representative pseudocolor plots show five subsets, including population 1 (P1: TCRβ^lo^CD69^lo^), population 2 (P2: TCRβ^int^CD69^lo^), population 3 (P3: TCRβ^int^CD69^hi^), population 4 (P4: TCRβ^hi^CD69^hi^), and population 5 (P5: TCRβ^hi^CD69^lo^) in donor-derived thymocytes from Control and *Srsf1* ^fl/fl^ *Cd4*-Cre mice, respectively. The frequency of indicated subsets was shown in **(C)** (n ≥ 3). **(D–H)** Analysis of the post-selection TCRβ^hi^ thymocytes from donor-derived mice. **(D)**TCRβ^hi^ thymocytes [populations 4 and 5 in **(B)**] were further fractionated into CD69^+^CD24^+^ immature and CD69^−^CD24^−^ mature subsets. **(F)** The immature subsets were subdivided into CD4^+^, CD4^+^CD8^lo^ (CD8^lo^), DP, and CD8^+^ sub-populations (clockwise from left in the top row), and the mature subsets were further subdivided into CD4^+^ and CD8^+^ populations (bottom row). The frequency of indicated subsets was shown in **(E, G)**, accordingly. **(H)** The ratio of CD4^+^ and CD8^+^ thymocytes was calculated and shown (n ≥ 3). Data are representative from at least two independent experiments. The error bars are means ± SD. Statistical significance was determined by one-tailed Student’s *t*-test. ns, not statistically significant; **P < 0.01 and ***P < 0.001.

To determine whether the developmental defects in *Srsf1* ^fl/fl^ *Cd4*-Cre were T cell autonomous, we generated bone marrow chimeric mice as described in **Figure 4A**. We found thymocytes derived from *Srsf1* ^fl/fl^ *Cd4*-Cre mice had a phenotype identical to that of thymocytes in primary SRSF1-deficient mice as described above (**Figures 4B**–**H**). The severe defects were detected in population 4 and 5 of thymocytes derived from *Srsf1* ^fl/fl^ *Cd4*-Cre mice (**Figures 4B, C**), and the frequency of TCRβ^hi^CD69^-^CD24^-^ mature population was substantially reduced (**Figures 4D, E**). In chimeric mice transplanted with *Srsf1* ^fl/fl^ *Cd4*-Cre donor cells, the frequency of donor-derived CD8^+^ SP cells was remarkably reduced in both TCRβ^hi^CD69^+^CD24^+^ immature and TCRβ^hi^CD69^-^CD24^-^ mature thymocytes, and the ratio of CD4^+^ to CD8^+^ SP cells was notably increased, accordingly (**Figures 4F**–**H**). These data thus demonstrated the impacts on maturation of late thymocytes and CD8 lineage fate were T cell intrinsic.

### SRSF1 Contributes to the Lineage Determination of CD4-Versus-CD8 Thymocytes

**Figure 5:**
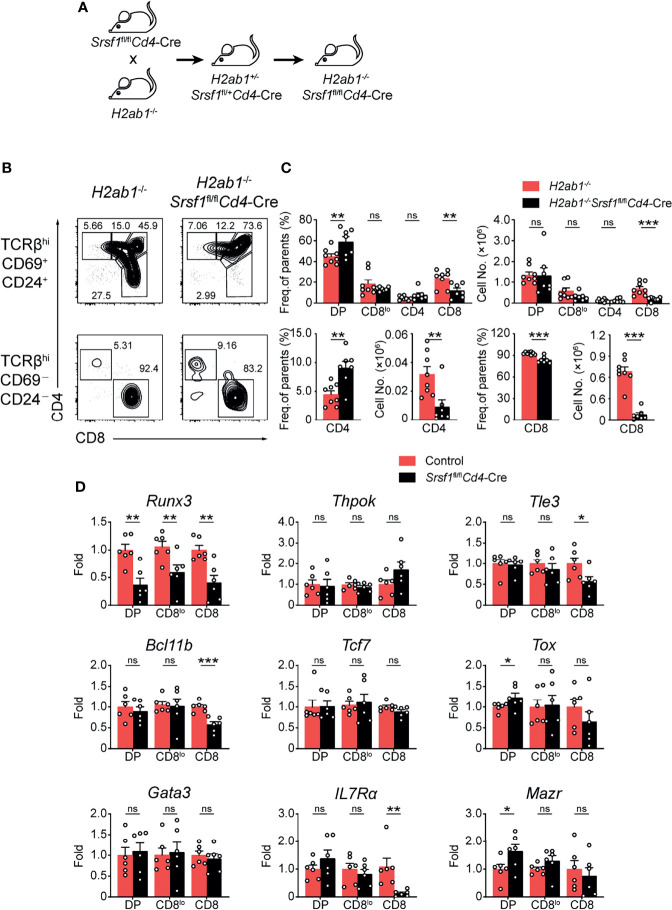
SRSF1 is involved in the lineage selection of CD4-versus-CD8 T cells. **(A)** The scheme shows the generation of *H2ab1* ^-/-^ *Srsf1* ^fl/fl^ *Cd4*-Cre mice. **(B, C)** Analysis of the post-selection TCRβ^hi^ thymocytes from *H2ab1* ^-/-^ *Srsf1* ^fl/fl^ *Cd4*-Cre mice. The immature (TCRβ^hi^CD69^+^CD24^+^) subsets were subdivided into CD4^+^, CD4^+^CD8^lo^, DP, and CD8^+^ sub-populations (clockwise from left in top row), and the mature (TCRβ^hi^CD69^−^CD24^−^) subsets were further subdivided into CD4^+^ and CD8^+^ populations (bottom row). The frequency and numbers of indicated subsets were shown in **(C)**, respectively (n ≥ 6). **(D)** Analyzing the expression of *Runx3*, *Thpo*k (*Zbtb7b*), *Tle3, Bcl11b, Tcf7, Tox, Gata3, IL7Rα* and *Mazr* in immature TCRβ^+^ DP, CD4^+^CD8^lo^, and mature CD8^+^ SP thymocytes from Control or *Srsf1* ^fl/fl^ *Cd4*-Cre mice. The relative expression of *Srsf1* transcript in indicated T cell subsets (after normalization to *Gapdh*) in Control cells was set as 1, and its relative expression in cells from *Srsf1* ^fl/fl^ *Cd4*-Cre mice was normalized, accordingly. Data were collected from at least two independent experiments. The error bars are means ± SD. Statistical significance was determined by one-tailed Student’s *t*-test. ns, not statistically significant; *P < 0.05, **P < 0.01, and ***P < 0.001.

To further evaluate how SRSF1 contributes to CD8^+^ lineage choice, we crossed *Srsf1* ^fl/fl^ *Cd4*-Cre mice with MHC class II-deficient (*H2ab1^-/-^*) mice, which lack mature CD4^+^ SP thymocytes (**Figure 5A**). We found the frequency of CD8^+^ SP cells in both immature and mature thymocytes from *H2ab1^-/-^Srsf1* ^fl/fl^ *Cd4*-Cre mice was substantially lower compared with those in their control mice (**Figure 5B**). The frequency of CD4^+^ SP cells in mature thymocytes from *H2ab1^-/-^Srsf1* ^fl/fl^ *Cd4*-Cre mice was significantly higher than those from their control mice (**Figure 5B**). The number of both mature and immature CD8^+^ SP cells was dramatically lower in *H2ab1* ^−^ *^/^* ^−^ *Srsf1* ^fl/fl^ *Cd4*-Cre mice, accordingly (**Figure 5C**). In contrast, the number of immature CD4^+^ SP cells was comparable from *H2ab1* ^−^ *^/^* ^−^ *Srsf1* ^fl/fl^ *Cd4*-Cre and Control mice, whereas the number of mature CD4^+^ SP cells was diminished in *H2ab1* ^−^ *^/^* ^−^ *Srsf1* ^fl/fl^ *Cd4*-Cre due to SRSF1 deficiency (**Figure 5C**). These data collectively indicated that SRSF1 deficiency impaired the CD8 lineage identity. We next detected the expression of genes involved in lineage selection in immature TCRβ^+^ DP, CD4^+^CD8^lo^, and mature CD8^+^ SP thymocytes, including *Runx3*, *Thpok (Zbtb7b)*, *Tle3, Bcl11b, Tcf7, Tox, Gata3, IL7Rα* and *Mazr*. The abundance of CD8 master regulator *Runx3* was substantially reduced in all three stages, and the significant elevation of *Tox* and *Mazr* was observed in DP stage but no changes in CD4^+^CD8^lo^ and mature CD8^+^ SP thymocytes in SRSF1-deficient cells (**Figure 5D**). Although the expression of *Tle3, Bcl11b*, and *IL7Rα* was dramatically decreased in CD8^+^ SP thymocytes, most of detected lineage commitment-related genes were not altered in the essential transient stages (DP and CD4^+^CD8^lo^), such as *Thpok, Tcf7, Tle3, Bcl11b*, and *Gata3* (**Figure 5D**). These results imply that SRSF1 may contribute to the CD8 lineage fate by primarily controlling *Runx3* expression.

### Overexpression of Runx3 Partially Rectify the Ratio of CD4^+^ to CD8^+^ Cells in *Srsf1* ^fl/fl^ *Cd4*-Cre Mice

**Figure 6:**
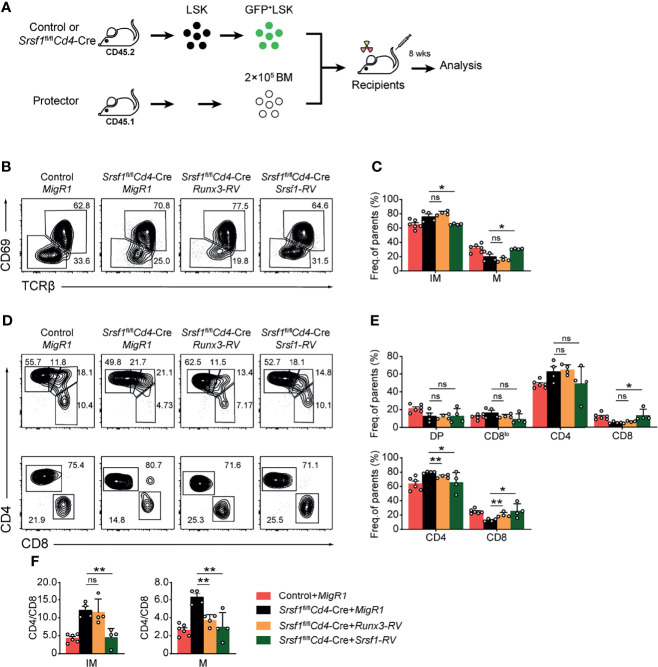
Overexpression of Runx3 partially rescues the defects of CD8^+^ T proportion in *Srsf1* ^fl/fl^ *Cd4*-Cre mice. **(A)** The flow chart shows the experimental design of bone marrow chimeric mice by using *Runx3*- or *Srsf1*-contained retroviral transduction. **(B)** Analysis the post-selection thymocytes from chimeric mice. TCRβ^hi^ thymocytes were further fractionated into CD69^+^CD24^+^ immature and CD69^−^CD24^−^ mature subsets. **(C)** The frequency of indicated subsets in **(B)** was shown, accordingly (n ≥ 4). **(D)** The immature subsets were subdivided into CD4^+^, CD4^+^CD8^lo^, DP, and CD8^+^ sub-populations (clockwise from top left in the top row), and the mature subsets were further subdivided into CD4^+^ and CD8^+^ populations (bottom row). **(E)** The frequency of indicated subsets in **(D)** was shown, respectively. **(F)** The ratio of CD4^+^ to CD8^+^ cells from immature and mature subsets of TCRβ^hi^ thymocytes was calculated and shown, accordingly (n ≥ 4). Data were collected from at least two independent experiments. The error bars are means ± SD. Statistical significance was determined by one-tailed Student’s *t*-test. ns, not statistically significant; *P < 0.05 and **P < 0.01.

We next attempted to explore whether enforced expression of Runx3 could rectify the defects in the CD8 lineage fate caused by SRSF1 deficiency. To achieve this goal, the retrogenic mouse models were established and analyzed as described in the flowchart (**Figure 6A**). We confirmed the transduced efficiency of BM LSK cells was more than 50% before transplantation (**Figure S3**) to ensure the successful construction of chimeric mice. By analyzing donor-derived TCRβ^hi^ post-selection thymocytes, we found that the reduction of mature (TCRβ^hi^CD69^−^CD24^−^) thymocytes was substantially restored by forced expression of SRSF1, but not by forced expression of Runx3 compared with those derived from Control-*MigR1* or *Srsf1* ^fl/fl^ *Cd4*-Cre-*MigR1* donors (**Figures 6B, C**). Meanwhile, the ectopic expression of SRSF1 also rectified the ratio of CD4^+^ to CD8^+^ SP cells in both TCRβ^hi^CD69^+^CD24^+^ immature and TCRβ^hi^CD69^−^CD24^−^ mature thymocytes (**Figures 6D**–**F**). However, overexpression of Runx3 could largely restore the ratio of CD4^+^ to CD8^+^ SP cells in TCRβ^hi^CD69^−^CD24^−^ mature stage while no rescue was observed in the TCRβ^hi^CD69^+^CD24^+^ immature stage (**Figures 6D**–**F**). These data collectively revealed that Runx3 serves as a regulator downstream SRSF1 for CD8 lineage fate decision, but other regulators and more complicated mechanisms may involve in the SRSF1-dependent regulatory network of late thymocyte maturation and lineage fate decision.

## Discussion

The lineage commitment of T cell to either CD8^+^ or CD4^+^ lineage before egress from thymus has been a fundamental research interest in the field of immunology, but the precise mechanism remains incompletely understood. Increasing evidences demonstrate that RBPs are indispensable for the development and function of immune cells by modulating gene expression through mRNA destabilization or stabilization, or by controlling translation (32–34), which provide a new direction to decode the complicated regulatory network in T cell fate decision. As a prototypical splicing factor, SRSF1 is well characterized for its roles in the maintenance of genomic stability, cell viability and cell-cycle progression (23, 35, 36), over the past twenty years, SRSF1 has been extensively investigated owing to its critical involvement in multiple cancers and autoimmune diseases (37–41). However, the roles of SRSF1 in T cell development and function have not been exposited until we recently found that it serves as a key posttranscriptional regulator in sustaining both the conventional T cell development and iNKT cell differentiation (28, 29).

As a follow-up study of the work by Qi et al. (28), we here report that conditionally targeting SRSF1 in DP thymocytes impairs the post selected T cell development and CD8^+^ T cell fate decision. Although previous study established the importance of SRSF1 in late thymocyte development and terminal maturation by using *Srsf1* ^fl/fl^ *Lck* ^Cre/+^ mice (28), the altered ratio of CD4^+^ to CD8^+^ cells has not been specifically addressed. To avoid the impacts caused by SRSF1 deletion at early stage, we employed *Srsf1* ^fl/fl^ *Cd4*-Cre mice to investigate the stage-specific role of SRSF1 in lineage choice in current study. We found the phenotypic defects were weaker in late stage of thymocyte development and maturation from *Srsf1* ^fl/fl^ *Cd4*-Cre mice than those from *Srsf1* ^fl/fl^ *Lck* ^Cre/+^ mice. Consistent with previous results from *Srsf1* ^fl/fl^ *Lck* ^Cre/+^ mice, the peripheral T cells were substantially decreased from *Srsf1* ^fl/fl^ *Cd4*-Cre mice, and most of the existing mature T cells were escapees in secondary lymphatic organ, which was caused by increased apoptosis and the shortened lifespan of SRSF1-deficient cells (28). Despite the substantial reduction of CD8^+^ SP cells was exhibited in both TCRβ^hi^CD69^+^CD24^+^ immature and TCRβ^hi^CD69^−^CD24^−^ mature thymocytes, the CD4^+^ SP cells were only notably reduced in TCRβ^hi^CD69^−^CD24^−^ mature stage, suggesting SRSF1 deficiency has more severe effects in CD8^+^ lineage differentiation.

To inspect whether SRSF1 contributes to the lineage choice of post selected DP thymocytes, we crossed the *Cd4*-Cre-mediated SRSF1 deletion mouse strain with the MHC-II-deficient *H2ab1* ^−/−^ mice. As expected, post selected mature thymocytes from control mice were largely restricted to the CD8^+^ T cell lineage because of the defective MHC-II expression. In contrast, mature thymocytes from *H2ab1* ^−/−^ *Srsf1* ^fl/fl^ *Cd4*-Cre mice contained fewer CD8^+^ SP cells but more CD4^+^ SP cells, indicating MHC-I–selected thymocytes are redirected from CD8^+^ to CD4^+^ T cell lineage in the absence of SRSF1. For potential targets involved in lineage choice and CD8 cell identity downstream SRSF1, we measured the well-established lineage commitment-related genes in three sequential developmental stages DP, CD4^+^CD8^lo^, and mature CD8^+^ T cells. In SRSF1-deficient cells, we found significant reduced expression of *Runx3* in three sequential developmental stages, and elevated expression of *Tox* and *Mazr* in only DP, but not CD4^+^CD8^lo^ stage, which is an essential transient population from DP thymocytes to either CD4^+^ or CD8^+^ SP cells (14). In addition, the expression of *Tle3, Bcl11b*, and *IL7Rα* was only reduced in CD8^+^ SP cells, which may miss the critical time point for lineage selection but affect the CD8 cell terminal maturation and survival. The dysregulation of lineage commitment-related genes leads to the aberrant differentiation of CD8^+^ SP thymocytes and jointly contributes to the abnormal ratio of CD4 to CD8 cells in *Srsf1* ^fl/fl^ *Cd4*-Cre mice, and Runx3 plays a central role downstream of SRSF1, particularly. However, overexpression of Runx3 could rectify the ratio of CD4^+^ to CD8^+^ SP cells in TCRβ^hi^CD69^−^CD24^−^ mature stage, but not completely rescue the defects in SRSF1-deficient mice, suggesting the complex mechanisms involved in the defective identity of CD8^+^ T cell in absence of SRSF1. Therefore, further understanding of how SRSF1 controls the expression of Runx3 as well as CD8 cell fate decision is required in future study.

In summary, our data revealed that SRSF1 exerts its developmental stage-specific effects in late thymocytes and contributes to CD8^+^ T cell lineage fate decision and identity. This study represents an important step to further decipher the physiological functions of SR proteins, providing new insights of RBPs in regulating T cell development and lineage commitment.

## Materials and Methods

### Animals

All mice used in this study were between 7 and 10 weeks of age on a fully C57BL/6J background. *Srsf1* ^fl/fl^ mice were kindly provided by Dr Xiang-Dong Fu (University of California, San Diego). *Cd4*-Cre and *H2ab1* ^−/−^ mice from Jackson Laboratories were maintained in the animal facility of China Agricultural University. Mice were housed in specific pathogen-free conditions under controlled temperature (22 ± 1°C) and exposed to a constant 12-hour light/dark cycle. All institutional and national guidelines for the care and use of laboratory animals were followed and all animal protocols used in this study were approved by the Institutional Animal Care and Use Committee at China Agricultural University.

### Flow Cytometry

Single cell suspensions obtained from thymus (Thy), spleen, lymph node (LN), and peripheral blood cells (PBCs) were stained with fluorochrome-conjugated antibody as described previously (42). The fluorochrome-conjugated antibodies listed below: CD4 (RM4-5), CD8a (53-6.7), CD24 (M1/69), CD69 (H1.2F3), TCRβ (H57-597), B220 (RA3-6B2), CD11b (M1/70), CD11c (N418), CD45.1 (A20), CD45.2 (104), CD49b (DX5), Gr.1 (RB6-8C5), TER119 (TER-119), TCRγδ (GL-3), ScaI (D7), cKit (2B8) and 7AAD (00-6993-50) were purchased from eBiosciences. The fluorochrome-conjugated streptavidin (554063) was purchased from BD Biosciences. Samples were acquired on a LSRFortessa or FACSVerse (BD Biosciences) and analyzed with FlowJo software v10.4.0 (Tree Star, Inc.). For cell sorting, cells were surface-stained with indicated fluorochrome-conjugated antibodies and subjected to sorting on a FACSAria II (BD Biosciences).

### Gene Expression Analysis

The gene expression was measured by qPCR as previously described (43). Briefly, total RNA was extracted from sorted cells using RNasey Mini Kit (Cat. # 74106, Qiagen) according to manufacturer’s instructions. FastQuant RT Kit (Cat. # KR106-02, Tiangen) was used to synthesize cDNA. Quantitative RT-PCR (qPCR) was performed with SYBR Green Master Mix (Cat. # FP205-02, Tiangen) using CFX96 Connect™ Real-Time System (Bio-Rad). The primers were shown in **Supplementary Table 1**. Fold differences in expression levels were calculated according to the 2^−ΔΔCT^ method and the relative expression of indicated genes was normalized to *Gapdh*.

### BM Chimeras

The BM chimeric mice were generated as previously described (44). Briefly, the lethally irradiated B6.SJL (CD45.1^+^) mice were transferred intravenously with a 1:1 mixture of 1 × 10^6^ BM cells from *Srsf1* ^fl/fl^ *Cd4*-Cre (CD45.2^+^) or control mice together with BM cells from congenic B6.SJL (CD45.1^+^) mice. After 10 weeks reconstitution, recipients were sacrificed and analyzed.

### Retroviral Transduction

The retrogenic chimera mouse models were generated by a modified protocol as previously described (28, 45). Briefly, retroviral packaging was carried out by transfection of HEK293T cells with *Runx3* cDNA bearing retroviral vector or empty pMigR1 vector along with pCLeco using Lipofectamine 2000 (Cat. # 11668019, Invitrogen), and the retrovirus-containing medium was collected at 24- and 48-hours post-transfection. After being filtered by 0.45 µm filters, the retrovirus-containing medium was loaded and centrifuged onto RetroNectin-coated [10 μg/mL (Cat. # T100A, TaKaRa)] non-tissue culture 24 well plates (Cat. # 351147, Falcon). BM cells from Control and *Srsf1* ^fl/fl^ *Cd4-*Cre mice were depleted of lineage positive cells and cultured for 24 hours in IMDM medium in the presence of thrombopoietin (20 ng/mL), stem cell factor (50 ng/mL), 15% FBS, 2-mercaptoethanol (50 µm), streptomycin and penicillin (100 μg/mL) in retrovirus contained RetroNectin plate as described above. Then, cells were infected with fresh retrovirus-containing medium in the presence of 8 μg/mL Polybrene (Cat. # H9268, Sigma-Aldrich) by centrifuging at 1,000 rcf for 90 min at 32°C. Subsequently, the cells were cultured for 2 hours at 37°C 5% CO_2_ incubator and resuspended in IMDM medium supplemented with components and cytokines as above. On the next day, the cells were spino-infected again. The infected cells were collected and analyzed by flow cytometry 24 hours later, and then these cells containing 5,000 GFP^+^ lineage^−^ScaI^+^cKit^hi^ (LSK) cells were transplanted into lethally irradiated (7.5 Gray) recipients (CD45.1^+^). The recipients were sacrificed to analyze at 8 weeks after transplantation.

### Gene Set Enrichment Assay

GSEA (v4.0.2) was used to analyze RNA-Seq data (GSE141349) from the GEO database, and the gene sets used in the article were obtained from MSigDB.

### Statistical Analysis

Statistical analysis was carried out through using GraphPad Prism software (version 8.0). Statistical significance was determined by one-tailed Student’s *t*-test. *, *P* < 0.05; **, *P* < 0.01; ***, *P* < 0.001.

## Data Availability Statement

The datasets presented in this study can be found in online repositories. The names of the repository/repositories and accession number(s) can be found in the article/**Supplementary Material**.

## Ethics Statement

The animal study was reviewed and approved by China Agricultural University Laboratory Animal Welfare and Animal Experiment Ethics Review Committee.

## Author Contributions

SYY designed the project and supervised the overall experiments. CJ, LB, and SZY performed the major experiments. CJ and MY analyzed the overall data and generated figures. MY analyzed the high throughput data. ZQ, FW, GY, JiL, XC, ZW, JuL, WG, MF, and FC assisted the overall experiments. SYY, YK, and CJ wrote the manuscript with the revision from all authors. All authors contributed to the article and approved the submitted version.

## Funding

This study was supported in part by grants National Natural Scientific Foundation of China (32130039, 31970831, 82170230, 31630038, and 81770207), National Key Research and Development Program of China (2017YFA0104401), the Chinese Universities Scientific Fund (2021TC087), the Project for Excellent Young Scientist of State Key Laboratory of Agrobiotechnology (2022SKLAB1-1), and the Project for Extramural Scientists of State Key Laboratory of Agrobiotechnology from China Agricultural University (2021SKLAB6-3, 2021SKLAB6-4, 2019SKLAB6-6, and 2019SKLAB6-7).

## Conflict of Interest

The authors declare that the research was conducted in the absence of any commercial or financial relationships that could be construed as a potential conflict of interest.

## Publisher’s Note

All claims expressed in this article are solely those of the authors and do not necessarily represent those of their affiliated organizations, or those of the publisher, the editors and the reviewers. Any product that may be evaluated in this article, or claim that may be made by its manufacturer, is not guaranteed or endorsed by the publisher.
