# Supplementary material for: SRSF1 Deficiency Impairs the Late Thymocyte Maturation and the CD8 Single-Positive Lineage Fate Decision
Source: Front Immunol. 2022 Jan 26;13:838719. doi: 10.3389/fimmu.2022.838719 (PMC8825371; doi:10.3389/fimmu.2022.838719)
Supplement: Supplementary file 1 [file DataSheet_1.pdf]

Supplementary Figure 1

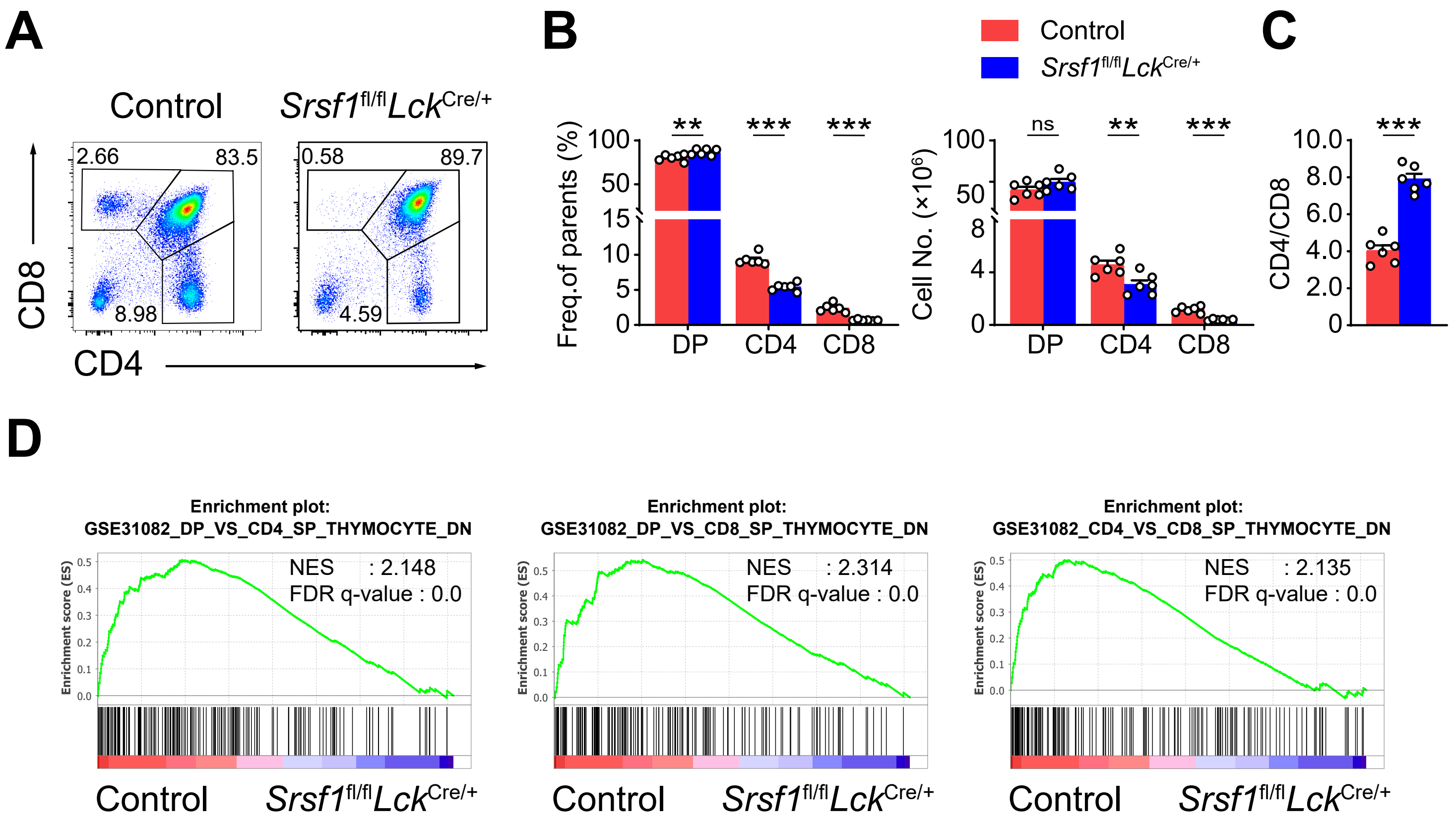

Figure S1. SRSF1 deficiency resulted in severe defects in CD8⁺ single positive thymocytes
(A) Flow cytometry analysis of thymocytes from *Srsf1*$^{fl/fl}$*Lck*$^{Cre/+}$ mice. Representative pseudocolor plots show the CD4⁺, CD8⁺, and CD4⁺CD8⁺ double positive (DP) thymocytes in *Srsf1*$^{fl/fl}$*Lck*$^{Cre/+}$ mice and their littermate controls (Controls).
(B) The frequency and numbers of indicated populations in (A) were shown, accordingly.
(C) The ratio of CD4⁺ to CD8⁺ thymocytes in (B) was calculated and shown as bar graph.
(D) Gene set enrichment analysis (GSEA) of signature genes of CD4⁺ or CD8⁺ T cells during development from DP thymocytes by using our existing data (GSE141349). Gene sets were supplied by MSIGDB from published data (GSE31802).
Data are representative from at least two independent experiments. The error bars are means ± standard deviation (SD). Statistical significance was determined by one-tailed Student's t-test. ns, not statistically significant; *P < 0.05, **P < 0.01, and ***P < 0.001.

Supplementary Figure 2

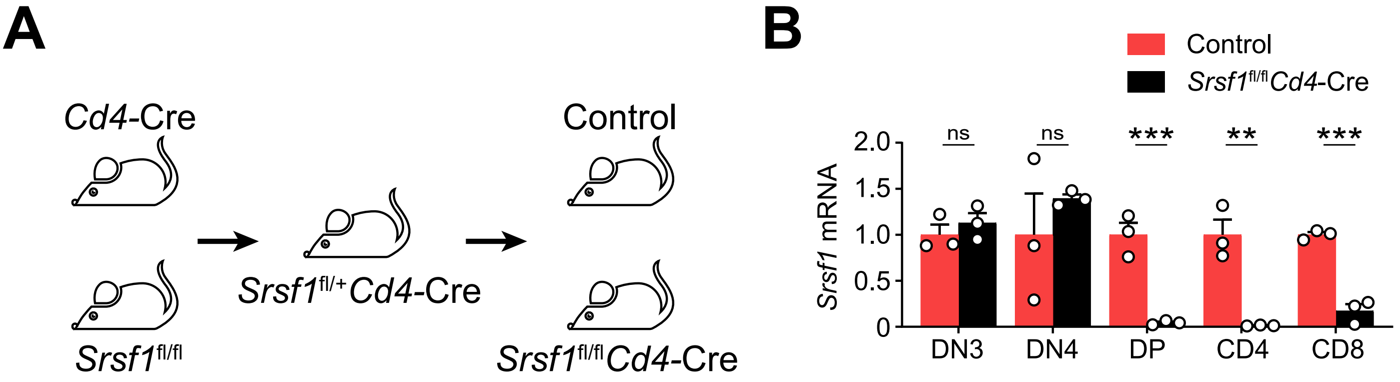

Figure S2. The establishment of *Srsf1*$^{fl/fl}$*Cd4*-Cre mouse model

(A) The scheme shows the breeding strategy of *Cd4*-Cre and *Srsf1*$^{fl/fl}$ mice to conditionally deactivate SRSF1 in DP thymocytes.

(B) Analysis of the deletion efficiency of *Srsf1* by quantitative RT-PCR (qPCR) in CD4$^-$CD8$^-$ double negative CD44$^-$CD25$^+$ (DN3), CD44$^+$CD25$^+$ (DN4), TCRβ$^+$CD4$^+$CD8$^+$ double positive (DP), TCR$^+$ CD4$^+$CD24$^-$ CD69$^-$ mature SP (CD4), and TCRβ$^+$ CD8$^+$CD24$^-$CD69$^-$ mature SP (CD8). The relative expression of *Srsf1* transcript in indicated T cell subsets (after normalization to *Gapdh*) in control cells was set as 1, and its relative expression in cells from *Srsf1*$^{fl/fl}$*Cd4*-Cre mice was normalized, accordingly.

Data are representative from at least two independent experiments. The error bars are means ± SD. Statistical significance was determined by one-tailed Student's t-test. ns, not statistically significant; *$P < 0.05$, **$P < 0.01$, and ***$P < 0.001$.

Supplementary Figure 3

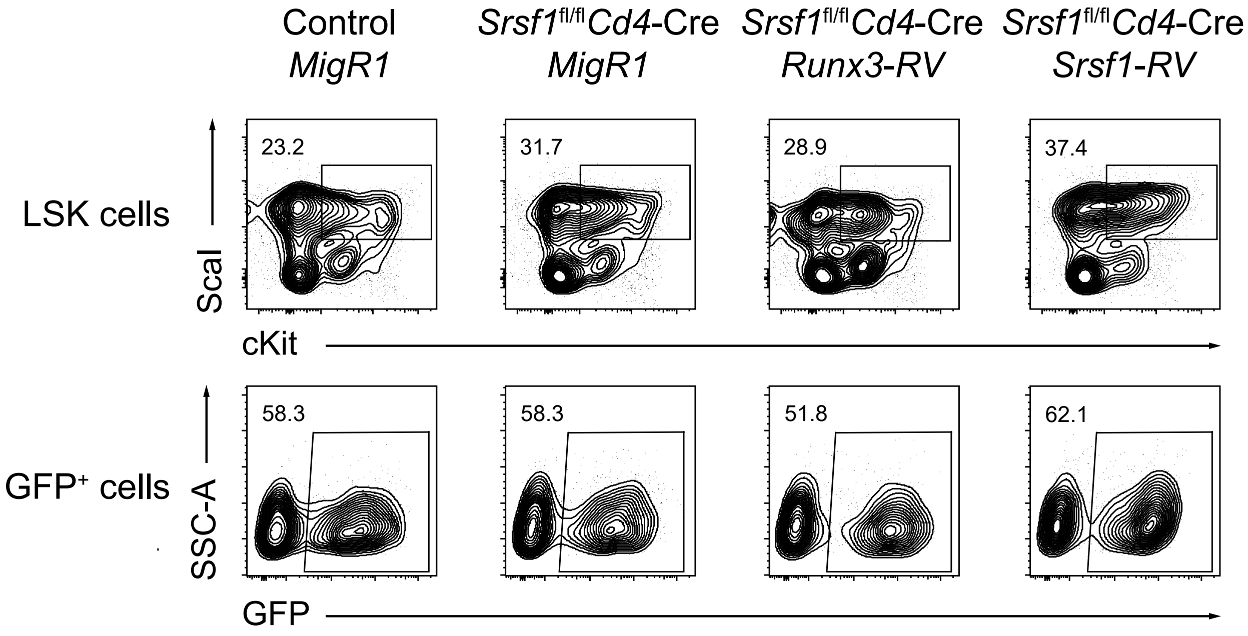

Figure S3. Detection of the transduced efficiency of lineage$^-$ScaI$^+$cKit^{hi} (LSK) cells before transplantation

The frequency of LSK was shown from collected cells infected by indicated retrovirus (top row). The frequency of GFP$^+$ LSK was analyzed for the calculation of donor cell number prior to transplantation (bottom row).
