# Supplementary material for: SRSF1 Deficiency Impairs the Late Thymocyte Maturation and the CD8 Single-Positive Lineage Fate Decision
Source: Front Immunol. 2022 Jan 26;13:838719. doi: 10.3389/fimmu.2022.838719 (PMC8825371; doi:10.3389/fimmu.2022.838719)
Supplement: Supplementary file 2 [file Table_1.docx]

Supplementary Table 1

Table S1. The primer list of qPCR

| **Name** | **Sequence 5’ → 3’** |
| --- | --- |
| **Primers for qPCR** | |
| *Bcl11b*-F | AGGAGAGTATCTGAGCCAGTG |
| *Bcl11b*-R | GTTGTGCAAATGTAGCTGGAAG |
| *Gapdh-*F | ATGGTGAAGGTCGGTGTGAA |
| *Gapdh-*R | GTCGTTGATGGCAACAATCTCC |
| *Gata3*-F | CTTATCAAGCCCAAGCGAAG |
| *Gata3*-R | CATTAGCGTTCCTCCTCCAG |
| *IL7Rα*-F | TACTTCAAAGGCTTCTGGAGCGAG |
| *IL7Rα*-R | ACGCCTTTCACCTCATGAATCTGG |
| *Mazr*-F | GCAGGTGCACACTTCTGAGCGAC |
| *Mazr*-R | GCATTTCTGGCCTTCTCGGTTACA |
| *Runx3*-F | AGGGAAGAGTTTCACGCTCA |
| *Runx3*-R | AGGCCTTGGTCTGGTCTTCT |
| *Srsf1*-F | TCTGCATGTCCTCTGTGTGAC |
| *Srsf1*-R | GCTCCCATCTGCAATTTAGC |
| *Tcf7*-F | CCCTTCCTGCGGATATAGAC |
| *Tcf7*-R | GGTACACCAGATCCCAGCAT |
| *Thpok*-F | CCCTGCTCGAGTTTGCTTAC |
| *Thpok*-R | CTCGCTCACAGTCATCCTCA |
| *Tle3*-F | ACACACCAACGCCAAGAAAT |
| *Tle3*-R | GGGATGGGATGTTGTGTAGG |
| *Tox*-F | GAGGATGCCTCCAAGATCAA |
| *Tox*-R | GCCTGGGTATCACGAAAGAA |
